# The correlation between urinary 5-hydroxyindoleacetic acid and sperm quality in infertile men and rotating shift workers

**DOI:** 10.1186/1477-7827-8-138

**Published:** 2010-11-08

**Authors:** Águeda Ortiz, Javier Espino, Ignacio Bejarano, Graciela M Lozano, Fabián Monllor, Juan F García, José A Pariente, Ana B Rodríguez

**Affiliations:** 1 Extremadura Centre for Human Assisted Reproduction, Badajoz, Spain; 2 Department of Physiology, Neuroimmunophysiology and Chrononutrition Research Group, Faculty of Science, University of Extremadura, Badajoz, Spain

## Abstract

**Background:**

Serotonin is a neurotransmitter that modulates a wide range of neuroendocrine functions. However, excessive circulating serotonin levels may induce harmful effects in the male reproductive system. The objective of this study was to evaluate whether the levels of urinary 5-hydroxyindoleacetic acid (5-HIIA), a major serotonin metabolite, correlate with different classical seminal parameters.

**Methods:**

Human ejaculates were obtained from 40 men attending infertility counselling and rotating shift workers by masturbation after 4-5 days of abstinence. Urinary 5- HIIA concentration was quantified by using a commercial ELISA kit. Forward motility was assessed by a computer-aided semen analysis (CASA) system. Sperm concentration was determined using the haemocytometer method. Sperm morphology was evaluated after Diff-Quik staining, while sperm vitality was estimated after Eosin-Nigrosin vital staining.

**Results:**

Our results show that urinary 5-HIIA levels obtained from a set of 20 volunteers negatively correlated with sperm concentration, forward motility, morphology normal range and sperm vitality. On the other hand, we checked the relationship between male infertility and urinary 5-HIIA levels in 20 night shift workers. Thus, urinary 5-HIIA levels obtained from 10 recently-proven fathers were significantly lower than those found in 10 infertile males. Additionally, samples from recent fathers exhibited higher sperm concentration, as well as better forward motility and normal morphology rate.

**Conclusions:**

In the light of our findings, we concluded that high serotonin levels, indirectly measured as urinary 5-HIIA levels, appear to play a role as an infertility determinant in male subjects.

## Background

Standard parameters evaluated, i.e. sperm morphology and motility, have shown different associations with male fertility. Particularly, Zhang et al. [1] demonstrated significant correlations among *in vitro *semen tests (total sperm concentration and motile sperm), zona pellucida-binding assay, and *in vitro *blastocyst production. On the other hand, the integrity of the plasma membrane reflects sperm viability and several process related to sperm physiology, such as capacitation, acrosome reaction, and binding of spermatozoa to the oocyte surface, require a biochemically active membrane [2,3]. Considering that capacitated and/or acrosome- reacted spermatozoa have a limited life span [4], this would result in impaired fertility.

The major function of the circadian system is the internal cycling of physiologic and metabolic events. Circadian rhythms are synchronized to the 24-h day, mostly by light-dark cycles, partially by other environmental and social time cues [5,6]. The circadian rhythm can get desynchronized in rotating shift workers since their night activity is out of phase, and such desynchronization may contribute to important health problems. For instance, the effects of rotating and night shift work on female fertility have been reported [7]. Additionally, undergoing night shift work may alter secretion circadian rhythms, including serotonin secretion [8].

Serotonin is a neurotransmitter involved in a wide range of behavioural and physiological processes. In fact, serotonergic neurons play an important role modulating neuroendocrine functions such as food intake, sleep, mood and sexual behaviour [9]. In the male reproductive system, serotonin might affect directly sperm maturation since the existence of a local serotonergic system in the rat caput epididymis have been described [10]. However, relatively recent studies have reported that hyperserotoninaemia may relate to certain kinds of male infertility [11,12]. In this regard, several studies have pointed out that selective serotonin reuptake inhibitors, which are commonly prescribed as antidepressants, can impair semen quality and damage sperm DNA integrity [13,14]. Therefore, up to now, the relationship between serotonin and male fertility still remains unclear. To this end, we aimed to evaluate whether the levels of urinary 5-hydroxyindoleacetic acid (5-HIIA), a major serotonin metabolite, correlate with different seminal parameters, including sperm concentration, motility and morphology, in a set of individuals attending infertility counselling. On the other hand, we checked the relationship between male infertility and urinary 5-HIIA levels in night shift workers by comparing 5-HIIA levels and seminal parameters between a group of recent fathers and another of infertile men, all of them undergoing night shift work.

## Methods

### Semen collection and preparation

Human semen was obtained from 40 men (20-40 year -old), as approved by the institutional review board of the University of Extremadura and the ethics committee of Infantile Hospital (Badajoz, Spain), as well as in accordance with the Declaration of Helsinki. Each subject was ascertained to be in good health by means of their medical histories and a clinical examination including routine laboratory test and screening. The subjects were all non smokers, were not using any medication and abstained from alcohol. Informed consent was obtained from all the participants. Samples were collected by masturbation after 4-5 days of sexual abstinence and allowed to liquefy (30 minutes, 37°C) before processing. All samples were collected at the same time of the year, thereby avoiding the seasonal and photoperiodic variations of sperm sample [15].

Routine seminal parameters were evaluated according to the World Health Organization criteria [16]. Thus, forward motility (grade a + b sperm motility) was assessed by a computer-aided semen analysis (CASA) system (Sperm Class Analyser, Microptic S.L., Spain). Sperm concentration, expressed as 10^6 ^cells/mL, was determined using the haemocytometer method on two separate preparations of the semen sample. Sperm morphology, measured as percentage of normal cells, was evaluated after Diff-Quik, while sperm vitality, assessed as percentage of viable cells, was estimated after Eosin-Nigrosin vital staining, which has been developed to evaluate sperm membrane integrity and let us distinguish intact, live spermatozoa from those that have lost their sperm membrane integrity (dead spermatozoa).

### Experimental design

The design of this study consisted of two parts. First of all, an *in vivo *study examined the link between urinary levels of 5-HIIA and sperm quality. Thus, we evaluated whether 5-HIIA levels measured in 20 volunteers correlated with different seminal parameters. These subjects were a mixture of infertile and fertile men. Normozoospermia was indicated by sperm concentration ≥ 20 × 10^6 ^cells/mL, progressive motility (grade a + b sperm motility) ≥ 50%, and normal sperm morphology ≥ 14%.

Secondly, we also examined whether serotonin, indirectly measured as urinary 5-HIIA, may influence sperm quality in 20 night shift workers. To this end, we compared the different seminal parameters between 10 recently-proven fathers, named as fertile group, and 10 infertile males, named as infertile group.

### Measurement of 5-hydroxyindoleacetic acid in urine

For urinary 5- HIIA determination, urines were collected at 19:00 h. The samples were stored at -20°C until biochemical assay. Urinary 5 -HIIA, which is a major serotonin metabolite, was quantified by using a commercial ELISA kit (IBL, Hamburg, Germany) according to the manufacturer's instructions. To adjust for variation in the dilution of urine, 5-HIIA concentrations were expressed as urinary 5- HIIA/urine creatinine. Creatinine concentration was determined by means of the Jaffe test, as described elsewhere [17].

### Statistical analysis

Pearson's correlation by multiple regression of different seminal parameters with the 5-HIIA levels were tested. Student's t-test was used at the significance level α = 0.05. Data displayed in histograms are expressed as mean ± SEM. All analyses were performed using GraphPad Prism^™ ^5 (GraphPad Software Inc., San Diego, CA, USA).

## Results and Discussion

**Figure 1:**
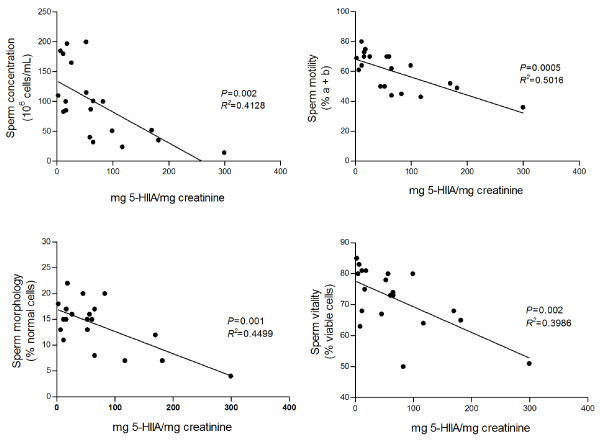
**Correlations between urinary 5-hydroxyindoleacetic acid (5-HIIA) levels and seminal parameters**. Urinary 5-HIIA levels found in urines (19:00 h) from 20 volunteers, assessed as mg 5-HIIA/mg creatinine, were correlated with different seminal parameters, including sperm concentration (10^6 ^cells/mL), forward motility (% a + b grades), morphology (% normal cells) and vitality (%).

Figure 1 shows the correlations between urinary 5-HIIA levels, obtained from 20 volunteers, and different seminal parameters analysed in fresh ejaculates. Curiously, urinary 5-HIIA levels negatively correlate with sperm concentration (statistically significant), forward motility (statistically significant), morphology normal range (statistically significant) and sperm vitality (statistically significant). Taken together, these findings point out that elevated urinary 5-HIIA levels may worsen sperm quality, contrary to our expectations.

**Figure 2:**
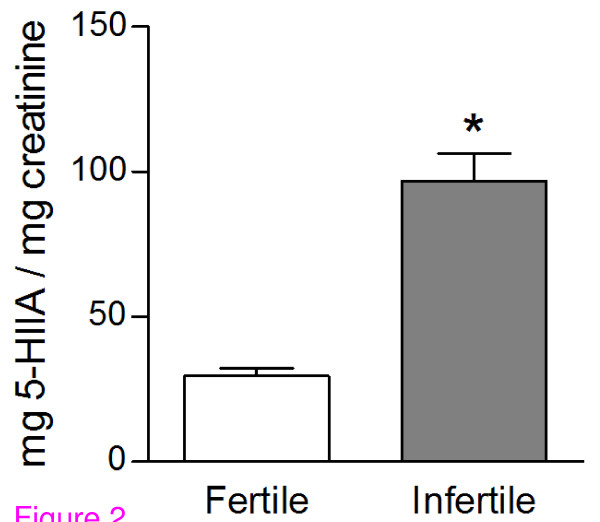
**Infertile night shift workers exhibit elevated 5- hydroxyindoleacetic acid (5-HIIA) levels**. Histograms display urinary 5-HIIA levels found in urines (19:00 h) from both fertile (recent fathers) and infertile night shift workers. Data are presented as mean ± SEM of 10 different volunteers. * statistically significant regarding controls.

To verify whether urinary 5-HIIA levels may influence sperm quality, we compared urinary 5-HIIA levels found in urines (19:00 h) from recently-proven fathers (n = 10), designed as fertile, with those obtained from infertile males (n = 10). All volunteers were working the night shift work, which is one of the facts that may alter serotonin circadian secretion [4]. Interestingly, urinary 5-HIIA levels measured in recently-proven fathers are lower (Figure 2, statistically significant) than those found in infertile males. In addition, we further investigated the relationship between serotonin and sperm quality by comparing different seminal parameters in both fertile and infertile night shift workers (Table 1). Our results display that samples from recently- proven fathers exhibit a higher sperm concentration (statistically significant), an increased forward motility (statistically significant), as well as an elevated normal morphology rate (statistically significant), compared with those values obtained in samples from infertile males. No differences were found with regards to sperm vitality (unpublished data). Therefore, the elevated serotonin endogenous levels, indirectly assessed as urinary 5-HIIA, may impair sperm quality, thus inducing male infertility in night shift workers.

**Table 1:** Seminal quality in night shift workers

Seminal Parameters	Fertile group (n = 10)	Infertile group (n = 10)
**Sperm concentration** **(106 cells/mL)**	148.0 ± 6.95	112.8 ± 3.35*
**Forward motility** **(% a + b grades)**	54.50 ± 1.04	39.67 ± 1.85*
**Morphology** **(% normal cells)**	22.80 ± 1.77	12.75 ± 1.10*

Data are presented as mean ± SEM of the number of volunteers

*statistically significant regarding fertile group

Male reproduction is likely controlled, at least partly, by circadian system. In fact, melatonin, an indoleamine whose production is subject to circadian rhythms with high levels at night and low by day [18], is able to modulate the reproductive physiology due to the melatonin regulator effect on the hypothalamus-pituitary-testicular axis [19]. Likewise, it has been reported that melatonin positively affects sperm physiology and may strongly protect spermatozoa from the oxidant environment produced by excessive round cells in seminal fluid [20,21].

Over the past few years, studies have proven that serotonin, another component of the circadian system, is necessary for the development of normal spermatogenesis in rats [22] owing to the existence of a local serotonergic system in the rat epididymis [10]. Nevertheless, as above-mentioned, high levels of serotonin appear to play a role as an infertility determinant in male subjects suffering from varicocele [12], or even azoospermy [23], i.e. an excess of circulating serotonin may induce harmful effects on male reproductive system. In fact, long-term treatments with antidepressants which enhance the availability of endogenous serotonin, such as sertraline or citalopram, can impair semen quality [13,14]. Despite both rats and human beings are mammals, we cannot assume that serotonin plays the same role on male reproductive system in both species because serotonin, which is also involved in sleep control [9], differently affects sleep/wake patterns in rodents and humans. In this sense, our findings shed some light on the relationship between serotonin and male infertility since we showed that the levels of urinary 5- HIIA negatively correlate with semen quality, as assessed by sperm concentration, motility or morphology.

## Conclusions

To sum up, elevated serotonin endogenous levels, indirectly assessed as urinary 5-HIIA, likely induce poor sperm quality that, consequently, leads to subfertility problems. In the light of these results, it is feasible to presume that a high dose of serotonin locally applied to the testicular surrounding area would be used as a reversible, cost-effective male contraceptive method. However, further studies are required to clarify molecular mechanisms through which serotonin modulates male reproductive system and to test whether serotonin supplementation has any noticeable side effect, such as sexual dysfunctions or mood changes.

## List of abbreviations

CASA: computer-aided semen analysis; 5-HIIA: 5-hydroxyindoleacetic acid.

## Competing interests

The authors declare that they have no competing interests.

## Authors' contributions

AO and JE carried out the experiments and drafted the manuscript. IB performed the statistical analysis and helped in drafting the manuscript. GML, FM and JFG collected sperm samples and performed the analysis of sperm parameters. JAP and ABR designed and conceived the study, interpreted the data and discussed the results. All authors read and approved the final manuscript.
